# Inorganic nanotubes reinforced polyvinylidene fluoride composites as low-cost electromagnetic interference shielding materials

**DOI:** 10.1186/1556-276X-6-137

**Published:** 2011-02-14

**Authors:** Varrla Eswaraiah, Venkataraman Sankaranarayanan, Sundara Ramaprabhu

**Affiliations:** 1 Alternative Energy and Nanotechnology Laboratory (AENL), Nano Functional Materials, Technology Centre (NFMTC), Department of Physics, Indian Institute of Technology Madras, Chennai 600036, India; 2 Low Temperature Physics Laboratory, Department of Physics, Indian Institute of Technology Madras, Chennai 600036, India

## Abstract

Novel polymer nanocomposites comprising of MnO_2 _nanotubes (MNTs), functionalized multiwalled carbon nanotubes (*f*-MWCNTs), and polyvinylidene fluoride (PVDF) were synthesized. Homogeneous distribution of *f*-MWCNTs and MNTs in PVDF matrix were confirmed by field emission scanning electron microscopy. Electrical conductivity measurements were performed on these polymer composites using four probe technique. The addition of 2 wt.% of MNTs (2 wt.%, *f*-MWCNTs) to PVDF matrix results in an increase in the electrical conductivity from 10^-16^S/m to 4.5 × 10^-5^S/m (3.2 × 10^-1^S/m). Electromagnetic interference shielding effectiveness (EMI SE) was measured with vector network analyzer using waveguide sample holder in X-band frequency range. EMI SE of approximately 20 dB has been obtained with the addition of 5 wt.% MNTs-1 wt.% *f*-MWCNTs to PVDF in comparison with EMI SE of approximately 18 dB for 7 wt.% of *f*-MWCNTs indicating the potential use of the present MNT/*f*-MWCNT/PVDF composite as low-cost EMI shielding materials in X-band region.

## Introduction

In recent years, electronics field has diversified in telecommunication systems, cellular phones, high-speed communication systems, military devices, wireless devices, etc. Due to the increase in use of high operating frequency and bandwidth in electronic systems, there are concerns and more chances of deterioration of the radio wave environment known as electromagnetic interference (EMI). This EMI has adverse effects on electronic equipments such as false operation due to unwanted electromagnetic waves and leakage of information in wireless telecommunications [1]. Hence, in order to maintain the electromagnetic compatibility of the end product, light weight EMI shielding materials are required to sustain the good working environment of the devices. EMI shielding refers to the reflection or absorption or multiple reflection of the electromagnetic radiation by a shielding material which thereby acts as a shield against the penetration of the radiation through it [2]. Conventionally, metals and metallic composites are used as EMI shielding materials as they have high shielding efficiency owing to their good electrical conductivity. Even though metals are good for EMI shielding, they suffer from poor chemical resistance, oxidation, corrosion, high density, and difficulty in processing [3]. The chemical resistance of polymer is defined largely by its chemical structure. In the present case, polyvinylidene fluoride (PVDF) has been chosen as the base polymer because of its excellent chemical resistance [4,5] over a variety of chemicals, acids, and bases. It is well known that the addition of lower amount of inorganic nanotubes (1-10 wt.%) will not affect the basic properties such as chemical resistance, strength, etc. of the base polymer [6,7]. Ever since the discovery by Ijima [8], carbon nanotubes (CNT) have attracted considerable research interest owing to their unique physical and chemical properties [9,10]. CNT-polymer composites gained popularity recently for various applications [11-13] due to the distinct advantages of polymers and nanofillers (CNT) such as lightweight, resistance to corrosion, and chemical resistance of the polymer as well as high electrical conductivity, high aspect ratio, and high mechanical strength of CNT [14,15].

Previous studies on CNT-polymer composites show that carbon nanotubes can be considered as advanced reinforcing materials possessing excellent electrical and mechanical properties and their unique one-dimensional structure [16,17] make them ideal for creating overlapping conductive network for high-performance EMI shielding at low loadings [18-21]. CNT-polymer composites either based on solvent casting or melt-based techniques have been studied with various polymer matrices, including PMMA [22], liquid crystal polymers, and melamine formaldehydes [23], PVA [24], and fused silica [25] for various applications such as radiation protection, EMI shielding, and electrostatic discharge materials. There are many reports on EMI shielding of carbon nanotubes reinforced polymer composites [26-30] in the X-band region because of its use in military communication satellites, weather monitoring, air traffic control, defense trackingand high-resolution imaging radars. But the disadvantage is the high loading of carbon nanotubes which is at present economically not feasible. So, there is a critical need for the development of low-cost EMI shielding materials at this particular frequency. Yonglai et al. [31] reported low-cost EMI shielding materials with the combination of carbon nanofiber and carbon nanotube composites in polystyrene (PS) matrix. They could achieve electromagnetic interference shielding effectiveness (EMI SE) of 20 dB for the combination of 10 wt.% carbon nanofiber and 1 wt.% carbon nanotubes in PS matrix in the range 12-18 GHz. In the present study, we have developed a low-cost hybrid EMI shielding material comprising of manganese dioxide nanotubes and low loading of multiwalled carbon nanotubes (MWCNTs) in PVDF matrix. EMI shielding efficiency and electrical conductivity of the composites with different weight fractions of functionalized multiwalled carbon nanotubes (*f*-MWCNTs) and MnO_2 _nanotubes (MNTs) were investigated to optimize polymer composites with less content of carbon nanotubes that exhibit enhanced electrical properties and serve as a better EMI shielding material. The focus of the present work is to fill the space between the MNTs using a low weight percent of *f*-MWCNTs within the polymer matrix and thereby making utmost use of the advantages of *f*-MWCNTs and eventually achieve low-cost and improved EMI shielding materials.

## Experimental section

### Materials

PVDF was used as polymer matrix with a molecular weight of 100,000 g.mol and it was purchased from Alfa Aesar. MWCNTs were synthesized by chemical vapor deposition technique. MNTs were prepared by hydrothermal route and *N*,*N*-dimethyl formamide was used as the solvent for carbon nanotubes and MnO_2 _nanotubes. Laboratory grade acids, bases, and organic solvents were used.

### Synthesis of functionalized multiwalled carbon nanotubes

MWCNTs were synthesized by chemical vapor deposition technique using misch metal (approximately 50% cerium and 25% lanthanum, with small amounts of neodymium and praseodymium)-based AB_3 _alloy hydride catalysts [32]. The as-grown MWCNTs not only contain pure MWCNTs but also amorphous carbon, fullerenes, and other metal catalysts. In order to remove these catalytic impurities and amorphous carbon, air oxidation was performed at 350°C for 4 h followed by acid treatment in concentrated HNO_3_. After purification, MWCNTs were functionalized with 3:1 ratio of H_2_SO_4 _and HNO_3 _at 60°C for 6 h in order to impart hydroxyl and carboxyl functional groups over the side walls.

### Synthesis of MnO_2 _nanotubes

MNTs were prepared by hydrothermal route [33]. Briefly, 0.608 g of KMnO_4 _and 1.27 ml of HCl (37 wt.%) were added to 70 ml of de-ionized water with continuous stirring to form the precursor solution. After stirring, the solution was transferred to a teflon lined stainless steel autoclave with a capacity of 100 ml. The autoclave was kept in an oven at 140°C for 12 h and then cooled down to room temperature. The resulting brown precipitate was collected, rinsed, and filtered to a pH 7. The as-prepared powders were then dried at 80°C in air.

### Synthesis of f-MWCNTs-MNTs-PVDF composites

MNTs and *f*-MWCNTs reinforced polymer matrix composites were prepared by mixing the respective composite solutions at high-speed rotations per minute followed by solvent casting. Here, we describe the method of preparation of the composites. Initially, 10 mg of MNTs and 990 mg of polymer were dispersed separately in dimethylformamide (DMF) with the help of an ultrosonicator for 1 h at room temperature for the preparation of 1 wt.% MNTs in polymer matrix. These two solutions were mixed by sonicating together for 1 h and the composite solution was transferred to a melt mixer and stirred at room temperature at 4,000 rpm for 2 h and at 80°C for 30 min. The resulting solution was transferred into the beaker and kept in an oven to remove the solvent. Finally, dried thin films were put in a mold and pressed to form 1-mm thick structures. A similar procedure was followed for the preparation of functionalized multiwalled carbon nanotubes (*f*-MWCNTs)/PVDF composite films. For the preparation of *f*-MWCNTs/MNTs/PVDF composite, fixed amount of MNTs, *f*-MWCNTs, and PVDF were added to DMF separately for a desired composition, and the above-mentioned procedure was followed to prepare the composite films. A series of composites were prepared in a similar way by varying the amount of polymer, MNTs, and MWCNTs.

### Characterization

**Figure 1:**
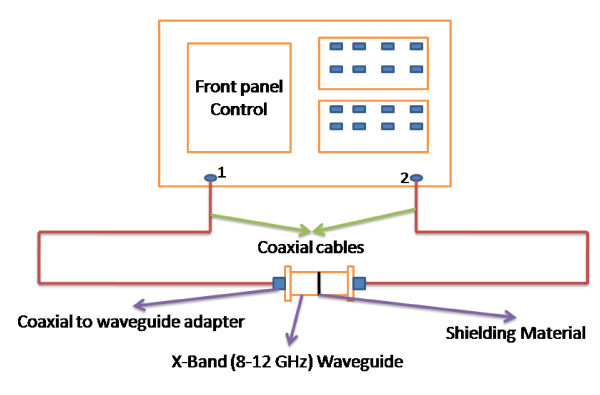
**Experimental setup for EMI shielding characteristic measurements of polymer composites**.

The direct current (DC) volume electrical conductivity of the composites was measured at room temperature using homemade resistivity setup with the help of Keithley 2400 sourcemeter and 2182 nanovoltmeter. The high resistance of the films was measured with a 617 programmable electrometer and a 6517B high-resistance electrometer. The EMI shielding measurement was performed with an Agilent E8362B vector network analyzer using a 201-point averaging in the frequency range of 8 to 12 GHz (X-band). Figure 1 shows the pictorial representation of the experimental setup for measuring the shielding effectiveness of the composite materials. Here, we followed the transmission line technique using an X-band waveguide sample holder for measuring scattering parameters of the composites. Samples of dimensions 22.84 × 10.16 mm^2 ^were prepared and kept inside the waveguide. The EMI shielding effectiveness is defined as the ratio of incoming (*P*_i_) to outgoing power (*P*_o_) of radiation. Shielding effectiveness (SE) = 10 log (*P*_i_/*P*_o_) and is defined in decibels (dB). The higher the value in decibels, the less energy passes through the material. When electromagnetic radiation falls on the shielding material, reflection, absorption, and transmission are observed. The corresponding reflectivity (*R*), absorptivity (*A*), and transmissivity (*T*) are according to the equation *A *+ *R *+ *T *= 1. *R *and *T *can be calculated from the measured scattering coefficients, from the relations S_12 _= 10 log *T *and *S*_11 _= 10 log *R*. The cross-sectional morphology of the composites were observed using field emission scanning electron microscope (FESEM, QUANTA 3 D, FEI) and transmission electron microscope. X-ray elemental mapping was also performed using EDX genesis software. Powder X-ray diffraction (XRD) studies were carried out using X'Pert PRO, PANalytical diffractometer with nickel filter Cu K_α _radiation as the X-ray source. The samples were scanned in steps of 0.016° in the 2*θ *range 10 to 80. For the determination of functional groups, a Fourier transform infrared spectrum was acquired using Perkin Elmer FTIR spectrometer from 400 to 4,000 cm^-1^. The chemical resistance of the composites in different acids, bases, alkanes and organic solvents was estimated by measuring the weight of the sample before and after treatment with these chemicals using METTLER TOLEDO XS 105 weighing balance.

## Results and discussion

### X-ray diffraction analysis

**Figure 2:**
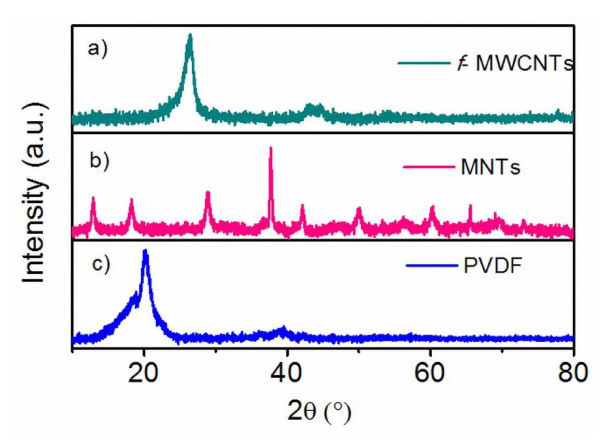
**X-ray diffractograms of *f*-MWCNTs, MNTs, and PVDF**.

The crystal structure of polymer, MNTs, and *f*-MWCNTs has been investigated by powder X-ray diffraction. Figure 2 shows the XRD pattern of the PVDF, *f*-MWCNTs, and MNTs. Figure 2a shows the XRD pattern of *f*-MWCNTs in which the peaks are indexed to the reflections of hexagonal graphite. The absence of additional peaks corresponding to the catalytic impurities confirms that the impurities have been removed by the acid treatment. The XRD spectrum of the as-synthesized MNT is shown in Figure 2b. All the diffraction peaks can be indexed according to the α-MnO_2 _phase, and no other characteristic peaks from any impurity are observed. This establishes the high purity of the sample. In Figure 2c, it can be seen that pure PVDF membrane is crystalline in nature with visible peaks at 18.65° and 20.09°. The sharp peak at 20.09° can be attributed to the presence of β-polymorph.

### Fourier transform infrared analysis

**Figure 3:**
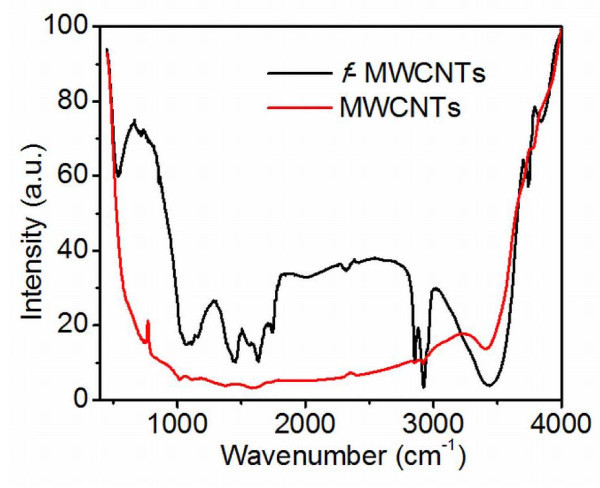
**FTIR spectra of purified and functionalized MWCNTs**.

Figure 3 shows the FTIR spectra of purified and functionalized MWCNTs (*f*-MWCNTs). The broad absorption band at 3,438 cm^-1 ^is attributed to the hydroxyl group (*ν*OH). The asymmetric and symmetric stretching of CH bonds are observed at 2,927 and 2,853 cm^-1^, respectively and the stretching of C = O of the carboxylic acid (-COOH) group is observed at 1,734 cm^-1^. The stretching of C = C, O-H bending deformation in -COOH and CO bond stretching in the *f*-MWCNTs are observed at 1,635 cm^-1^; 1,436 cm^-1^; and 1,073 cm^-1^; respectively indicating that carboxyl and hydroxyl functional groups were attached to the surface of MWCNTs.

### Raman spectra analysis

**Figure 4:**
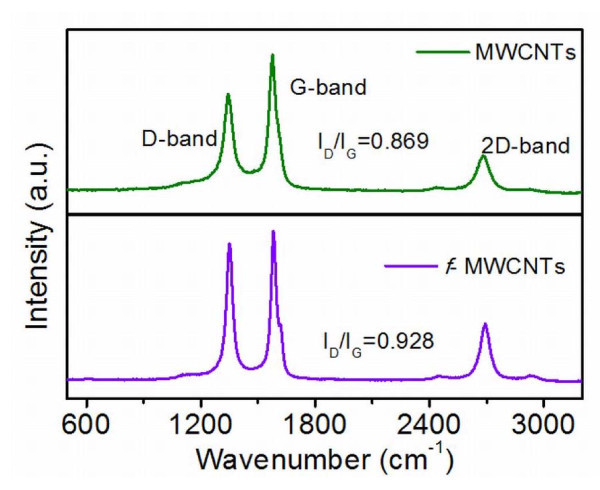
**Raman spectra of purified and functionalized MWCNTs**.

Figure 4 shows the Raman spectra of purified and functionalized MWCNTs. The spectra consists of three main peaks. The peak at 1,343 cm^-1 ^is assigned to the defects and disordered graphite structures, while the peaks at 1,586 cm^-1 ^and 2,693 cm^-1 ^are attributed to the graphite band which is common to all sp^2 ^systems and second-order Raman scattering process, respectively. Intensity ratio of defect band and graphite band is a signature of the degree of functionalization of the MWCNTs. As seen from Figure 4, I_D_/I_G _of pure carbon nanotubes is 0.868 whereas that for functionalized carbon nanotubes is 0.928 indicating the more defective nature of *f*-MWCNTs.

### Morphology and composition analysis

**Figure 5:**
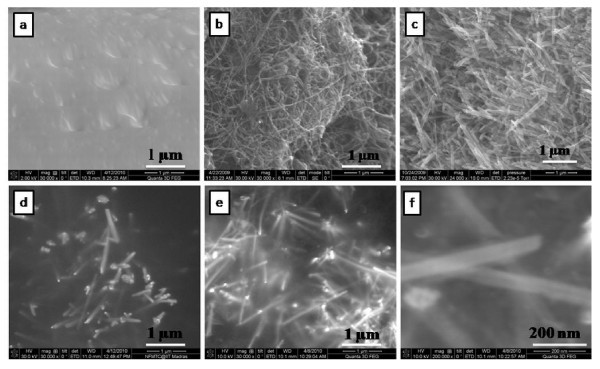
**Field emission scanning electron microscope images**. **(a) **PVDF, **(b) ***f*-MWCNTs, **(c) **MNTs, **(d) **1 wt.% MNTs-PVDF, **(e) **2 wt.% MNTs-PVDF, and **(f) **high-resolution image of 2 wt.% *f*-MWCNTs-PVDF.

**Figure 6:**
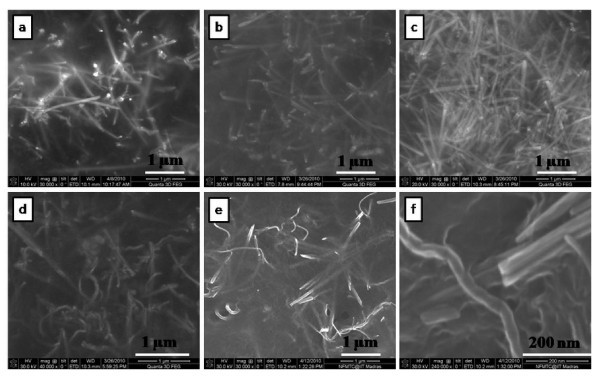
**Field emission scanning electron microscope images**. **(a) **3 wt.% MNTs-PVDF, **(b) **4 wt.% MNTs-PVDF, **(c) **5 wt.% MNTs-PVDF, **(d) **1 wt.% *f*-MWCNTs-5 wt.% MNTs-PVDF, and **(e) **2 wt.% *f*-MWCNTs-5 wt.% MNTs-PVDF, and **(f) **high-resolution image of 1 wt.% *f*-MWCNTs-5 wt.% MNTs-PVDF.

**Figure 7:**
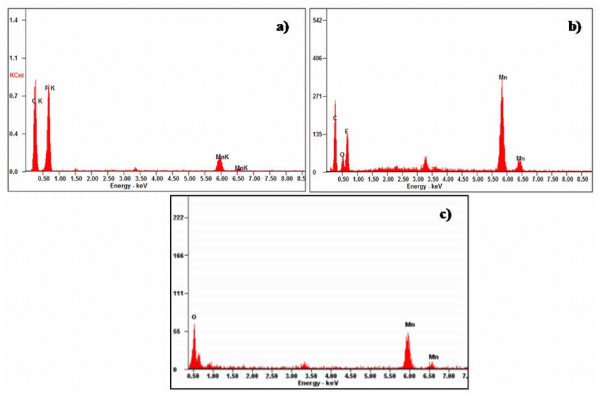
**Energy dispersive X-ray spectra of MnO_2 _nanotubes and its composites**.

**Figure 8:**
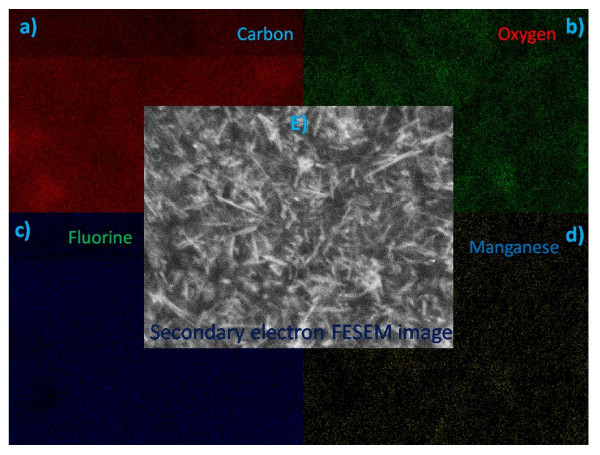
**X-ray elemental mapping of 5 wt.% MNT-1 wt.% *f*-MWCNTs-PVDF composite**.

Morphology is an important factor which affects the EMI SE of the composites. Figure 5a, b, c, d, e, f shows the FESEM images of polymer, nanofillers and nanofiller reinforced polymer composites. The corresponding images are (a) pure PVDF, (b) *f*-MWCNTs, (c) pure MNTs, (d) 1 wt.% MNTs-PVDF composite, (e) 2 wt.% MNTs-PVDF composite, and (f) high resolution image of 2 wt.% MNTs-PVDF composite. As shown in the Figure 5b andc, MWCNTs are 30 to 40 nm in diameter and approximately 10 μm in length and MNTs are 50 to 70 nm in diameter and in micron length. It can be observed that MWCNTs are entangled with each other because of Van der Waals interactions, whereas manganese dioxide nanotubes were straight and rigid and PVDF shows smooth surface as shown in the Figure 5a. *f*-MWCNTs and MNTs were homogeneously distributed and embedded in the PVDF matrix as shown in Figure 6a, b, c, d, e, f due to ultrasonication and shear mixing of the solutions at high rpm in the formation of composite films. Figure 6d, e, f indicates that the space between filler aggregates in carbon nanotube-PVDF composites is much smaller than that of MNTs-PVDF composites. Figure 6e shows the FESEM image of 5 wt.% MNTs filled PVDF composite along with 1 wt.% MWCNTs. It is observed that a very good microstructure has been formed, and *f*-MWCNTs were uniformly dispersed and embedded between the MNTs throughout the PVDF matrix. This good network can increase the number of inter nanostructure connections, and hence provide better EMI SE. Further, to confirm the homogeneity of the composites, we have performed X-ray elemental mapping over the sample surface to visualize the atomic elements of manganese, oxygen, carbon, and fluorine. Figure 7 shows the EDX spectra of PVDF-based MNTs and *f*-MWCNTs composite. It confirms the presence of manganese and oxygen from MnO_2_, carbon from *f*-MWCNTs, and fluorine from the PVDF polymer. Figure 8 shows the elemental mapping of the 5 wt.% MNTs-1 wt.% *f*-MWCNTs-PVDF composite. As can be seen from the figures, all the elements were distributed homogeneously in the polymer matrix.

### Chemical resistance of the polymer composites

The percentage of chemical resistance of the composites in different acids, bases, organic solvents, and alkanes are shown in the Table 1. It indicates that all the polymer composites are highly resistant towards the chemicals. The MNT-MWCNTs-PVDF composite shows 95% to 100% resistance towards chemicals which indicates the potentiality of the present composite. For comparison, the chemical resistances of MWCNT-PVDF, PVDF, and MNT-PVDF composites were also measured.

**Table 1:** Percentage of chemical resistance for different polymer composites

Chemical	Percentage of chemical resistance			
	**PVDF**	**5 wt.% MNT-*f*-MWCNT-PVDF**	**1 wt.% *f*-MWCNT-** **PVDF**	**5 wt.% MNT-** **PVDF**

Acetic acid glacial	98.9	97.9	98.2	98.0

Oleic acid	100	100	98.5	100

Sodium hydroxide solution	97.6	96.0	98.8	97.2

Ammonia solution	98.7	98.4	95.7	96.8

n-Hexane	97.8	100.0	98.7	100

2-Propanol	98.9	100.0	98.3	97.3

Toluene	97.0	98.1	97.0	97.2

Chloroform	100	97.9	100	96.8

### Electrical conductivity analysis

**Figure 9:**
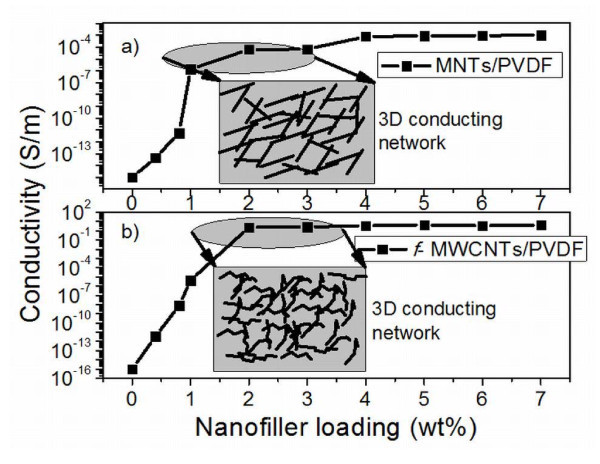
**DC electrical conductivity of the MNTs/PVDF and *f*-MWCNTs/PVDF composites**.

Electrical conductivity is of utmost importance for effective EMI shielding material. As shown in the Figure 9, the conductivity of the PVDF is about 10^-16^S/m. As the concentration of the MNTs increases in the PVDF matrix, electrical conductivity increases, and it follows percolation behavior. Conductivity of the 1 wt.% MNTs/PVDF composite was found to be approximately 10^-6^S/m, which indicates that there is a drastic improvement in electrical conductivity. An increase of about ten orders of magnitude of electrical conductivity was observed which can be attributed to the high aspect ratio and efficient dispersion of the MNTs in the PVDF matrix. Similar trend is observed in the case of electrical conductivity of the *f*-MWCNTs/PVDF composites as shown in Figure 9b. The possible mechanism for the increment in the electrical conductivity of the composites can be the tunneling effect of the electrons from one nanotube to the other. The effect of *f*-MWCNTs content on the electrical conductivity of the MNTs/PVDF composites was studied. Incorporation of 1 wt.% *f*-MWCNTs in 5 wt.% MNT/PVDF composites increases the conductivity from 10^-5^S/m to approximately 10^-1^S/m which can be attributed to the high aspect ratio, homogeneous dispersion, and high electrical conducting nature of the *f*-MWCNTs.

### Electromagnetic interference shielding effectiveness

**Figure 10:**
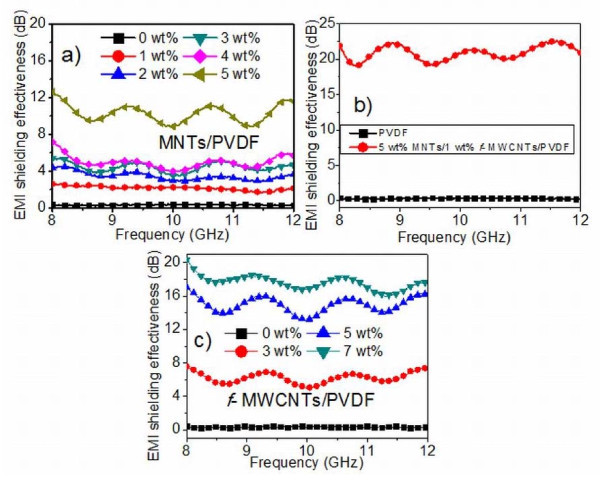
**EMI shielding effectiveness of MNTs/PVDF, MNTs/*f*-MWCNTs/PVDF and *f*-MWCNTs/PVDF composites**.

The EMI SE of MNTs/PVDF composites with various mass fractions of MNTs as a function of frequency are presented in Figure 10a. The results show that EMI shielding effectiveness of pure PVDF is almost 0.3 dB indicating that it is transparent to the electromagnetic radiation throughout the measured frequency. This is probably due to its electrically insulating nature. It is observed that EMI SE starts increasing with the addition of MNTs to the insulating PVDF matrix. The EMI SE for 1 wt.% MNTs filled PVDF composite is found to be 2.27 dB and it increases further to 5.14 and 11 dB at higher loading of MNTs of 3 and 5 wt.%, respectively. Hence, it is clear that the major contribution to the EMI shielding comes from the addition of semiconducting MNTs to the PVDF matrix. This increment in EMI SE can be attributed to the formation of conductive and connective network in the PVDF matrix, which is in accordance with the high-resolution FESEM image of MNTs filled PVDF composite (Figure 5f). Since electrical conductivity of the MNTs is two orders less compared to that of carbon nanotubes, there is a limit over the highest obtainable conductivity of the total composite. This limits the EMI SE to approximately 12 dB for 5 wt.% MNTs/PVDF composite. These results suggest that the MNTs/PVDF composites can be used for electrostatic discharge applications. In order to make it suitable for EMI shielding applications, a small amount of (1 wt.%) *f*-MWCNTs have been incorporated in MNT/PVDF matrix. With this, 1 wt.% *f*-MWCNTs in 5 wt.% MNTs/PVDF composite, we could achieve an EMI SE of 18 to 22 dB. For comparison, the EMI SE of 7 wt.% *f*-MWCNTs/PVDF composites alone in the same frequency region has been measured, and in this case, an EMI SE of 18 dB has been obtained as shown in Figure 10c. Table 2 shows the overall EMI SE of different composites and their electrical conductivities. It is clear that 5 wt.% MNTs-1 wt.% *f*-MWCNTs-PVDF composite can be a better and low-cost EMI shielding material.

**Table 2:** Electrical conductivity and EMI SE of the polymer composites

Composite	Electrical conductivity (S/m)	EMI SE (dB)
1 wt.% *f*-MWCNTs/PVDF	Approximately 10^-10^	Approximately 2

2 wt.% *f*-MWCNTs/PVDF	Approximately 10^-1^	Approximately 7

5 wt.% MNTs/PVDF	Approximately 10^-5^	Approximately 11

7 wt% *f*-MWCNTs/PVDF	Approximately 10^-1^	Approximately 18

5 wt.% MNTs/1 wt.% *f*-MWCNTs/PVDF	Approximately 10^-1^	Approximately 21

5 wt.% MNTs/2 wt.% *f*-MWCNTs/PVDF	Approximately 10^-1^	Approximately 20

### Shielding mechanism in MNTs/*f*-MWCNTs/PVDF composites

It is well reported that reflection is the most prominent EMI shielding mechanism in CNT-polymer composites [34]. In the present case, EMI shielding in *f*-MWCNTs reinforced PVDF composites has been studied and from the measured scattering parameters reflectivity, transmissivity, and absorptivity were derived using the formulae mentioned in the experimental section. For 5 wt.% *f*-MWCNT-PVDF composites, the transmissivity, reflectivity, and absorptivity are 0.177, 0.601, and 0.222, respectively and the corresponding parameters for 7 wt.% *f*-MWCNT-PVDF composites are 0.131, 0.794, and 0.075. From these results, we can conclude that reflection is the major EMI shielding mechanism in the present *f*-MWCNT-PVDF composites. This may be due to the presence of conjugated π electrons on the surface of *f*-MWCNTs. In the case of MNTs/PVDF composites, the chances of absorbing incident radiation are more due to the presence of electric dipoles. Table 3 gives a comparison of the reflectivity and absorptivity of various composites. It is observed that *f*-MWCNTs/MNTs/PVDF composites and MNTs/PVDF composites exhibit more absorption than reflection. For 5 wt.% MNTs/1 wt.% *f*-MWCNTs/PVDF composite, the absorptivity, transmissivity, and reflectivity values are respectively 0.78, 0.01, and 0.210. Based on the measured fundamental properties of MNTs/PVDF, *f*-MWCNTs/PVDF, and MNTs/*f*-MWCNTs/PVDF composites, the present composites can be engineered for reflection to absorption of the incoming EM radiation by varying the amount of carbon nanotubes and MnO_2 _nanotubes in the polymer matrix. The incorporation of MNTs in *f*-MWCNT-PVDF composite helps in overcoming the Van der Waals forces between *f*-MWCNTs while utilizing the high aspect ratio of them. Another advantage of the addition of MNTs is that it could decrease the amount of *f*-MWCNT loading in PVDF matrix.

**Table 3:** Transmissivity, reflectivity, and absorptivity of MNTs/*f*-MWCNTs/PVDF composites

Composite	Absorptivity	Transmissivity	Reflectivity
1 wt.% *f*-MWCNTs/PVDF	0.042	0.631	0.327

2 wt.% *f*-MWCNTs/PVDF	0.218	0.199	0.583

5 wt.% *f*-MWCNTs-PVDF	0.222	0.177	0.601

7 wt.% *f*-MWCNTs-PVDF	0.075	0.131	0.794

5 wt.% MNT-PVDF	0.530	0.1	0.370

7 wt.% MNT-PVDF	0.608	0.1	0.292

5 wt% MNT-1 wt.% *f*-MWCNTs-PVDF	0.780	0.01	0.210

7 wt% MNT-1 wt.% *f*-MWCNTs-PVDF	0.796	0.01	0.194

## Conclusion

Novel hybrid nanofiller consisting of multiwalled carbon nanotubes and MnO_2 _nanotubesreinforced PVDF composite has been fabricated and proposed as an efficient material for EMI shielding applications. MNTs and *f*-MWCNTs acting as spacers in PVDF matrix helps in reducing the aggregation of the nanofillers and creates an excellent 3 D conducting network in the polymer. MNTs are acting as very good filler material when added to the entangled carbon nanotubes incorporated polymer. An EMI shielding effectiveness of approximately 20 dB has been achieved with 5 wt.% MNTs and 1 wt.% *f*-MWCNTs in polymer matrix in X-band region. The increase in EMI shielding effectiveness with the addition of nanofillers is attributed to the enhanced electrical conductivity of the composite due to the addition of *f*-MWCNTs and good homogeneity of the nanofillers in the polymer. The present hybrid polymer nanocomposites are proposed as low-cost and efficient EMI shielding materials in X-band region.

## Competing interests

The authors declare that they have no competing interests.

## Authors' contributions

VER carried out the composites preparation, other characterizations and written the manuscript. VSN and SRP are conceived in its coordination. All authors read and approved the final manuscript.
